# Invasive fungal infections in a pediatric hematology-oncology department: A 16-year retrospective study

**DOI:** 10.18502/CMM.6.2.2840

**Published:** 2020-06

**Authors:** Nikoleta Kazakou, Timoleon Achilleas Vyzantiadis, Anastasia Gambeta, Eleni Vasileiou, Eleni Tsotridou, Dimitrios Kotsos, Athina Giantsidi, Anna Saranti, Maria Palabougiouki, Maria Ioannidou, Emmanuil Hatzipantelis, Athanasios Tragiannidis

**Affiliations:** 1 nd Department of Pediatrics, School of Medicine, Aristotle University of Thessaloniki, Thessaloniki, Greece; 2 First Department of Microbiology, School of Medicine, Aristotle University of Thessaloniki, Greece

**Keywords:** *Aspergillosis*, Children, Hematologic malignancies, Invasive candidiasis, Invasive fungal infections

## Abstract

**Background and Purpose:**

Invasive fungal infections (IFIs) are a major cause of morbidity and mortality in immunocompromised children. The purpose of our study was to evaluate the incidence of IFIs in pediatric patients with underlying hematologic malignancies and determine the patient characteristics, predisposing factors, diagnosis, treatment efficacy, and outcome of IFIs.

**Materials and Methods:**

For the purpose of the study, a retrospective analysis was performed on cases with proven and probable fungal infections from January 2001 to December 2016 (16 years)

**Results:**

During this period, 297 children with hematologic malignancies were admitted to the 2nd Pediatric Department of Aristotle University of Thessaloniki, Greece, and 24 cases of IFIs were registered. The most common underlying diseases were acute lymphoblastic leukemia (ALL; n=19,79%), followed by acute myeloid leukemia (AML; n=4, 17%) and non-Hodgkin lymphoma (NHL; n=1,4%). The crude incidence rates of IFIs in ALL, AML, and NHL were 10.5%, 18.2%, and 2.8% respectively. Based on the results, 25% (n=6) and 75% (n=18) of the patients were diagnosed as proven and probable IFI cases, respectively. The lung was the most common site of involvement in 16 (66.7%) cases. Furthermore, *Aspergillus* and *Candida* species represented 58.3% and 29.1% of the identified species, respectively. Regarding antifungal treatment, liposomal amphotericin B was the most commonly prescribed therapeutic agent (n=21), followed by voriconazole (n=9), caspofungin (n=3), posaconazole (n=3), micafungin (n=1), and fluconazole (n=1). In addition, 12 children received combined antifungal treatment. The crude mortality rate was obtained as 33.3%.

**Conclusion:**

As the findings of the present study indicated, despite the progress in the diagnosis and treatment of IFIs with the use of new antifungal agents, the mortality rate of these infections still remains high.

## Introduction

Patients with hematologic malignancies appear to be at a high risk of developing IFIs [ 1 ]. Children with cancer or primary/secondary immunodeficiency and those undergoing hematopoietic stem cell transplantation are the major subsets of pediatric patients at the risk of IFI development [ 1 - 6 ]. Prolonged and severe neutropenia, the administration of broad-spectrum antibiotics, cytotoxic chemotherapy, surgery, presence of a central venous catheter, and the use of total parenteral nutrition and corticosteroids increase the risk of IFIs [ 4 , 7 - 10 ]. Although in the last decades, there has been an improvement in the survival and outcome of children with cancer secondary to advances in preventive measures and supportive care, IFIs remain a devastating problem and are still associated with a high rate of morbidity and mortality [ 10 - 14 ].

The review of the international literature indicates a need for further published data on IFIs in pediatric patients with malignancies, especially for those not receiving primary antifungal prophylaxis. Therefore, early diagnosis and prophylactic empirical and preemptive antifungal therapy are important for prevention and treatment as reported in the pediatric international guidelines [ 15 , 16 ]. The revised definitions of invasive fungal disease proposed by the European Organization for Research and Treatment of Cancer/Invasive Fungal Infections Cooperative Group and the National Institute of Allergy and Infectious Diseases Mycoses Study Group (EORTC/MSG) retain the classifications of proven, probable, and possible IFIs [ 17 ]. 

In addition to the progress in diagnostics, the implementation of intensive monitoring, administration of prompt and effective treatments, avoidance of drug toxicity, use of appropriate surgical treatment where indicated, and minimization of delay in commencing chemotherapy are important measures in the management of IFIs [ 2 - 4 , 10 , 12 , 13 ]. With this background in mind, the aim of the present retrospective analysis was to report data regarding the proven and probable cases of IFIs in the Pediatric Oncology Unit of the 2nd Pediatric Department of the Aristotle University of Thessaloniki, Thessaloniki, Greece, from January 1, 2001 to December 31, 2016.

## Materials and Methods

This retrospective study was conducted on children with hematologic malignancies (e.g., acute/chronic leukemias and lymphomas), aplastic anemia, myelodysplastic syndromes, and solid tumors with ≤ 18 years of age at the time of diagnosis who underwent therapy at the Pediatric Oncology Unit of the 2^nd^ Pediatric Department of the Aristotle University of Thessaloniki (i.e., AHEPA Hospital) between January 1, 2001 and December 2016. The University AHEPA Hospital is an academic tertiary care hospital in the city of Thessaloniki in Northern Greece. The 2nd Pediatric Department is a pediatric unit that consists of the General Pediatric Clinic (50 beds) and Pediatric Hematology-Oncology Clinic (20 beds). The Pediatric Hematology-Oncology Clinic treats children and adolescents (<18 years old) with benign and malignant hematologic diseases and solid tumors. However, there is no hematopoietic stem cell transplantation unit in this hospital.

Patient characteristics were recorded with particular attention to medical history, underlying diseases, previous anticancer therapy (e.g., chemotherapy and corticosteroids), and current disease status (i.e., induction, remission, relapse, and refractory disease). The collected data included general characteristics, clinical presentation, predisposing factors, pathogens, antifungal treatment, type of therapy, and outcome. The patients were considered to have a proven or probable disease according to the international consensus criteria published in 2008 [ 17 ]. 

According to the EORTC/MSG consensus criteria, the patients were defined as having a proven IFI in case of the presence of a positive biopsy or positive microbiology from sterile body sites. On the other hand, those patients who presented with a host factor, clinical features, and mycological evidence were classified as having a probable IFI according to the previously mentioned EORTC/MSG consensus criteria [ 11 ]. Crude IFI incidence with a 95% confidence interval was calculated by dividing the number of oncology patients diagnosed with an IFI by the total number of patients seen for each oncology subtype during the same period. Furthermore, neutropenia was defined as an absolute neutrophil count of < 500 μL, and fever was defined as a single axillary-equivalent temperature of 38.5oC measured during a 6-hour period within at least 1-hour intervals.

The administration of chemotherapeutic agents prior to the episode of IFI was also recorded. The use of each antibiotic was recorded from commencement until the development of IFI. According to the international guidelines and local standard operating procedures, all episodes of febrile neutropenia were empirically treated with ceftazidime and amikacin until 2012. However, within 2012-2016, these cases were managed with piperacillin-tazobactam or meropenem with/without amikacin. In case of a hemodynamically unstable or persistent fever for longer than 72 h, a glycopeptide was empirically added. Empiric antifungal treatment was initiated in case of persistent fever after 72-96 h, and a thorax computed tomography (CT) was performed, in addition to sinus CT, if there was a suspicion of fungal sinusitis. 

Galactomannan test was introduced to our local practice in 2008 and performed prior to the initiation of empirical antifungal treatment twice per week in case of possible, probable, or proven invasive aspergillosis (IA). Antifungal prophylaxis was generally applied to the children defined as high risk for IFIs (i.e., those with AML, relapsed acute leukemia, and high-risk ALL) and varied over time. Empiric antifungal treatment with liposomal amphotericin B (L-AmB) or caspofungin was applied to all patients with persistent fever (>72 h) and unresponsive to empiric antibiotics. Toxicity was defined as organ dysfunction temporarily associated with the use of antifungal agents rather than other factors. Moreover, the efficacy of antifungal treatment was assessed by the evaluation of clinical, radiological, and mycological (laboratory) response and survival.

**Ethical considerations**

The authors confirm that the ethical policies of the journal have been adhered to. 

## Results

During a period of 16 years, 297 children with hematologic malignancies were admitted to the 2^nd^ Pediatric Department of Aristotle University of Thessaloniki, Greece. Out of this population, 24 children were enrolled and analyzed for proven and possible IFIs. A total of 24 IFIs were identified in 24 children. Table 1 tabulates data related to patient characteristics, underlying diseases, type of IFIs, site of infection, and antifungal treatment.

**Table 1:** Patient characteristics, underlying disease, type of invaive fungal infection, site of infection, antifungal treatment and outcome

Underlying disease	Age/gender	Treatment phase	Neutropenia (ANC< 500 /μl)	Site of IFI	Year of the IFI diagnosis	EORTC/MSG classification	Fungal species	Antifungal treatment	Outcome
ALL	13/F	Induction	Yes	Lung, spleen	2004	Probable	*Candida*	LAmB, voriconazole	Survival
ALL	2/F	Induction	Yes	Lung, CNS, spleen, GI	2003	Probable	*Candida*	LAmB, caspofungin	Death
ALL	2/M	Induction	No	Lung, CNS	2002	Probable	*Aspergillus*	LAmB	Survival
AML	9/F	Induction	Yes	Lung, sinus	2004	Proven	*Aspergillus*	LAmB, voriconazole	Death
ALL	8/F	Relapse	No	CNS	2004	Probable	*Aspergillus*	Voriconazole	Death
ALL	13/F	Induction	Yes	Lung, CNS	2008	Probable	*Aspergillus*	LAmBS	Survival
ALL	5/M	Induction	No	CNS	2005	Proven	*Cryptococcus neoformans*	LAmB, fluconazole	Death
AML	2/F	Induction, relapse	Yes	Lung	2002	Probable	*Aspergillus*	LamB	Survival
ALL	5/M	Induction	Yes	Lung	2009	Probable	*Aspergillus*	LAmB	Survival
AML	8/F	Induction, relapse	Yes	Lung	2010	Probable	*Aspergillus*	LAmB	Death
ALL	4/F	Induction	No	GI	2011	Probable	*Candida*	Voriconazole	Survival
NHL	13/M	Induction	No	GI, derma (trunk)	2010	Probable	*Candida*	LAmB	Survival
ALL	7/M	Induction	No	Lung	2013	Probable	*Aspergillus*	LAmB	Survival
ALL	6/M	Induction	Yes	Lung	2014	Probable	*Aspergillus*	LamB	Survival
AML	2/F	Relapse	Yes	Lung	2002	Probable	*Aspergillus*	LAmB, caspofungin	Death
ALL	2/M	Induction	Yes	Lung, spleen	2002	Probable	*Candida*	LAmB, caspofungin, voriconazole	Survival
ALL	5/M	Induction	Yes	Lung	2007	Probable	*Aspergillus*	Voriconazole	Survival
ALL	8/F	Induction	Yes	Lung	2007	Probable	*Aspergillus*	LAmB, voriconazole	Survival
ALL	13/F	Induction	Yes	Extremity (forearm)	2011	Proven	*Mucor*	LAmB,	Survival
ALL	3.5/F	Induction	Yes	Spleen,	2012	Proven	* Candida tropicalis*	LAmB, voriconazole, posaconazole	Survival
ALL	13/M	Relapse	Yes	Lung, derma (trunk)	2015	Probable	*Aspergillus*	LAmB, voriconazole	Death
ALL	3/M	Induction	Yes	Eye, zygomatic bone	2016	Proven	*Rhizopus Arrhizus*	LAmB, posaconazole	Survival
ALL	4/F	Induction	Yes	Lung	2014	Probable	*Aspergillus*	LAmB, voriconazole	Survival
ALL	9/M	Relapse	Yes	Blood	2012	Proven	* Candida albicans*	LAmB, micafungin	Death

**Demographic characteristics**

The study population consisted of 11 (45.8%) males and 13 (54.2%) females, with the mean age of 6.64 years (range: 2-13 years).

**Underlying condition**

The most common underlying condition was ALL (n=19,73%), followed by AML (n=4,17%) and NHL (n=1, 3%). The crude incidence rates of IFI in the patients with ALL, AML, and NHL were 10.5%, 18.2%, and 2.8%, respectively. In children with hematologic malignancies, 20 episodes of IFIs occurred after induction therapy in ALL cases or during the first 2 months of intensive chemotherapy in AML and NHL cases. 

**Site of infection**

A pulmonary infection was detected in 16 patients (66.7%), while other infection sites included the spleen (n=4, 16.7%), central nervous system (CNS; n=5, 20.5%), gastrointestinal tract (n=3, 12.5%), sinus (n=1), eye (n=1), skin (n=2), forearm (n=1), and bloodstream (n=1). Furthermore, Out of 24 patients, 9 cases (37.5%) had multiple sites of infection as reported in Table 1.

**Diagnostic findings**

According to the revised EORTC/MSG criteria, 6 of the diagnoses were classified as proven (25%). Among this group, 1, 1, 2, and 2 patients developed cryptococcosis (CNS cryptococcoma), IA (sinus/nasal aspergillosis), mucormycosis, and invasive* Candida* infection (chronic disseminated candidiasis and candidemia), respectively. Moreover, 18 (75%) of 24 cases presented with an IFI during or after an episode of severe neutropenia (ANC<500/mm^3^/duration of febrile neutropenia>10 days). 

**Treatment outcome**

Regarding the antifungal treatment, 12 (50%) patients received monotherapy with L-AmB and voriconazole with a survival rate of 83.3% (10/12). Combined antifungal treatment was administered to the remaining 12 patients due to showing unfavorable responses to conventional antifungal monotherapy and had a survival rate of 50%. The patients with mucormycosis were successfully treated with a combination of surgery and prolonged antifungal treatment with L-AmB and posaconazole. However, three patients with proven chronic disseminated candidiasis, cryptococcosis, and invasive aspergillosis passed away. One of the patients with candidiasis due to *Candida tropicalis* survived. In total, fluconazole, micafungin, posaconazole, caspofungin, voriconazole, and L-AmB were administered to 1, 1, 2, 3, 9, and 21 patients, respectively. Case fatality rate was 33%, which was higher for children with IA (35.7%).

## Discussion

treatment of IFIs, they still remain a major cause of morbidity and mortality in immunocompromised children [ 3 , 8 , 9 , 11 - 13 ]. The incidence rate of IFIs in pediatric oncology patients varies in different studies. Bartlett et al., performing a retrospective 10-year multicenter study on 337 cases of IFIs in 320 immunocompromised children in Australia, reported the crude prevalence rates of 10.6% and 28.2% in patients with ALL and AML, respectively [ 11 ]. In addition, Wang et al. presenting data from a retrospective 10-year multicenter study with 123 IFIs in 119 children with ALL and Bal et al. performing a retrospective 8-year study on 125 children with IFIs and underlying ALL reported the prevalence rates of 9.7% and 19.2%, respectively [ 12 , 13 ]. Our results revealed a similar trend as we documented a crude IFI incidence rate of 10.5% in our patients with ALL. As for children with AML, the incidence rate (18.2%) was slightly lower in our study, compared to the rate reported by Bartlett et al. Nonetheless, this could be related to the smaller number of patients with AML.

Regarding the spectrum of fungal infections, *Aspergillus* species was identified or an *Aspergillus* infection was documented in most of the cases (14/24, 58.3%). Furthermore, a* Candida* species was isolated, or invasive candidiasis/candidemia was diagnosed in 7/24 (29.2%) cases. In addition, Mucor was the etiological agent in 2/24 (8%) cases. Although invasive candidiasis continues to represent the most common IFI in patients with hematologic malignancies, our data demonstrated a high incidence of IA, probably associated with prolonged and severe neutropenia and the higher number of patients with hematologic malignancies admitted to our department. Another possible factor for the lower incidence of candidiasis/candidemia could be the wide use of prophylaxis in high-risk patients with fluconazole and voriconazole. Wang et al. also reported an ascendency of mold pathogens (65.9%), with *Aspergillus* species being the most common agent in children with ALL [ 12 ]. Bartlett et al. reported an equivalent prevalence of yeast and mold infections, with *Aspergillus* species being the main causative agent of mold infections. However, in the mentioned study,* Candida* species accounted for the majority of yeast infections (90.3%) [ 11 ]. In contrast to our findings, Bal et al. reported the detection of* Candida* species in 76.4% of children [ 13 ]. 

Out of 24 patients, 21 (87.5%) cases developed IFIs after undergoing induction therapy for hematologic malignancies. Kumar et al., Villarroel et al., and Mor et al. reported the rates of 22.9%, 5.8%, and 7.2%, respectively [ 18 - 20 ]. These differences can be attributed to the difference in the number of patients each study enrolled, as well as the different malignancies and chemotherapy regimens each unit administered. Among the IFIs detected in our study, the rates of proven and probable cases were obtained as 25% and 75%, respectively. The percentage of proven IFIs in pediatric patients varies widely in the literature. In a later study performed by Hovi et al., the incidence of IFIs was obtained as 3.2%, and this extremely low rate was probably due to the renovation of the ventilation system and initiation of routine azole antifungal prophylaxis. In other studies, the incidence of IFIs varies from 17.6% to 45.5% [ 11 , 12 , 18 , 20 , 21 ]. The differences reported in the percentage of proven IFIs may reflect the different prophylactic measures and strategies adopted by various centers and variations in diagnostic timelines and procedures. Febrile neutropenia, as a severe side effect of chemotherapy that raises mortality, is a key factor for accurate prognosis. Kumar et al. reported neutropenia in all patients, while Mor et al. obtained a profound neutropenia rate of 89%, along with IFI [ 18 , 20 ]. 

Concerning the treatment of IFIs, L-AmB, azoles, and echinocandins have been the main drugs in our armamentarium in the last 2 decades [ 22 - 24 ]. The simultaneous use of combined antifungal treatment has not met clinical approval or any support from current guidelines. However, in many cases, including ours, multi-drug treatment was administrated due to clinicians’ fear of systemic spread and the fact that IFIs are accompanied by high morbidity and can delay chemotherapy. In our study, half of the patients (out of 24 cases) were treated with one antifungal medication (i.e., either L-AMB or voriconazole), and the other half were administered with a combination of two or three antifungal medications. 

The crude mortality rate described in our retrospective study was 33.3%, which is comparable to the rates reported in other studies. In the current study, mortality was higher than the values obtained by Mor et al. (21.7%), Bartlett et al. (19%), Villaroel et al. (19%), Kumar et al. (9.4%), and Wang et al (4.2%)[ 11 , 12 , 18 - 20 ]. It should be noted that rates vary in different studies, depending on such factors as underlying disease or patient characteristics. Τhe mortality rates are still very high, which suggests that there is an imperative need for both early detection and effective treatment of IFIs in children with hematologic malignancies. 

The present study entails a number of limitations. The main limitation is that this study was conducted retrospectively and at a single center. Furthermore, there appears to be the heterogeneity of data regarding the causative agents. It should be also noted that the antifungal prophylaxis and treatment for aspergillosis and candidiasis varied during the 15-year period of our study. 

## Conclusion

As concluded in our retrospective study, IFIs are a problem mainly in children with hematologic malignancies but not solid tumors. The mortality rate of IFIs in immunocompromised patients remains high despite our armamentarium, which consists of new antifungal agents, such as echinocandins and newer azoles, as well as the improvement of the diagnostic process. It can be assumed that the diagnostic driven approach for IFIs, in contrast to the empirical driven treatment, may have an impact on the prompt diagnosis and initiation of the appropriate treatment. Nevertheless, it should be underlined that our study provides a valuable insight into IFIs epidemiology and outcomes in a large pediatric hematology-oncology center in Greece as it is the first study addressing this field in Greece.

## Author’s contribution

N. K. wrote the first draft of the manuscript, T. A. V. collected mycology data, A. G. and E. V. conceived, designed, and coordinated the research, and E. T., D. K., A. G, and A. S. collected all data retrospectively. M. P. and M. I. performed the critical revision, E. H. contributed to the study concept, and A. T. contributed to the study concept and managed the project. All authors revised the manuscript and contributed to improve the paper. All authors read and approved the final manuscript.

## Conflict of Interest:

The authors declare no conflicts of interest.

## Financial disclosure

The authors declare no financial disclosure.
